# Insights into Aldehyde Dehydrogenase Enzymes: A Structural Perspective

**DOI:** 10.3389/fmolb.2021.659550

**Published:** 2021-05-14

**Authors:** Kim Shortall, Ahmed Djeghader, Edmond Magner, Tewfik Soulimane

**Affiliations:** Department of Chemical Sciences, Bernal Institute, University of Limerick, Limerick, Ireland

**Keywords:** aldehyde dehydrogenase, NAD(P) cofactor, structure-function, mutations, enzyme dysfunction, oligomerization, C-terminal extensions, spirosomes

## Abstract

Aldehyde dehydrogenases engage in many cellular functions, however their dysfunction resulting in accumulation of their substrates can be cytotoxic. ALDHs are responsible for the NAD(P)-dependent oxidation of aldehydes to carboxylic acids, participating in detoxification, biosynthesis, antioxidant and regulatory functions. Severe diseases, including alcohol intolerance, cancer, cardiovascular and neurological diseases, were linked to dysfunctional ALDH enzymes, relating back to key enzyme structure. An in-depth understanding of the ALDH structure-function relationship and mechanism of action is key to the understanding of associated diseases. Principal structural features 1) cofactor binding domain, 2) active site and 3) oligomerization mechanism proved critical in maintaining ALDH normal activity. Emerging research based on the combination of structural, functional and biophysical studies of bacterial and eukaryotic ALDHs contributed to the appreciation of diversity within the superfamily. Herewith, we discuss these studies and provide our interpretation for a global understanding of ALDH structure and its purpose–including correct function and role in disease. Our analysis provides a synopsis of a common structure-function relationship to bridge the gap between the highly studied human ALDHs and lesser so prokaryotic models.

## Introduction

Aldehyde dehydrogenases (ALDH) (EC 1.2.1.3) are a family of nicotinamide adenine dinucleotide (phosphate) (NAD(P)) dependent enzymes, typically with a molecular mass of ca. 50–60 kDa. They oxidise a large range of aliphatic and aromatic, endogenous and exogenous aldehydes to form the corresponding carboxylic acids. They differ in their subcellular location, tissue distribution and preferred substrates while contributing to a broad spectrum of associated biological activities across prokaryotes, eukaryotes and Archaea. ALDHs are involved in detoxification, biosynthesis, antioxidant functions and structural and regulatory mechanisms (Vasiliou and Nebert, 2005). Interestingly, some if not most, ALDHs have multiple functionality. For example, human ALDH1A1, ALDH2, ALDH3A1, and ALDH4A1 can carry out esterase activity Sládek (2003), with ALDH2 also possessing nitrate reductase activity Sydow et al. (2004), suggesting more than one catalytic function of the ALDH family.

Unlike many other systems, ALDH investigation originated in human models, rather than bacterial ones which likely arises from their central link with pathological conditions. To date 10 out of the 19 human ALDHs have a resolved structure. Initially this led to misconceptions and a rather inaccurate description of typical features related to the enzyme family (Rodríguez-Zavala et al., 2006; Hayes et al., 2018). Emerging research allows for an accurate understanding of diversity within the ALDH family, across prokaryotes and eukaryotes, while bridging the gap to form a global perception of the superfamily. In addition, new enzyme structures demonstrating novel characteristics, provides scope for an updated, complete summary of the ALDH family structure to date.

ALDHs are generally grouped according to sequence identity, phylogeny and structural features combined, with the system evolving through identification of new superfamily members over time. Initial classification of ALDHs commenced in the late 1980s when only few ALDH sequences were available and which led to their classification into 3 distinct classes: ALDH 1, 2 and 3 Lindahl and Hempel (1990), Lindahl (1992), generally composed of human or mammalian isozymes. Classes 1 and 3 consist of both constitutively expressed and inducible cytosolic enzymes, with class 3 being expressed specifically in tumor, stomach and corneal cells. Class 2, contains constitutive mitochondrial enzymes. Each class oxidises a variety of substrates that can be derived from either an endogenous or exogenic source, including aldehydes derived from xenobiotic metabolism (Lindahl, 1992). Class 1 and 2 ALDHs contain mostly substrate variable enzymes while class 3 contains substrate specific ones (Perozich et al., 1999b). Generalisation with regards to the different classes has proposed that class 1 and 2 members, usually homotetramers, contain 500 amino acids per monomer with 70% sequence identity. Class 3 members, on the other hand, contain 450 amino acids, harboring an N-terminal deletion, and are of homodimeric nature (Rodríguez-Zavala et al., 2006). The sequence identity of class 3 in relation to class 1 or class 2 is only 25% (Perozich et al., 1999a). With a growing number of ALDH sequences, a new classification based on Dayhoff’s work Dayhoff (1976) was adopted later in 1999 to establish rules for ALDH nomenclature. Proteins with sequence identity greater than 40% were considered to belong to the same class, while proteins with more than 60% sequence identity have been assigned to the same sub-class (Vasiliou et al., 1999). ALDH nomenclature now spans from ALDH1 to ALDH24 across organisms (Brocker et al., 2013; Hou and Bartels, 2015). Supporting this, the human ALDH family consists of 19 putatively functional genes encoded on distinct chromosomal locations. However, emerging research demonstrates that significantly more ALDH classes exist. A recent study on the *Pseudomonas* genus identified 42 different classes of ALDHs demonstrating the scope of this enzyme’s diversity, but housed in a general, common structure (Riveros-Rosas et al., 2019).

## General Structure

### Introduction to the Aldehyde Dehydrogenase Common Architecture

**FIGURE 1:**
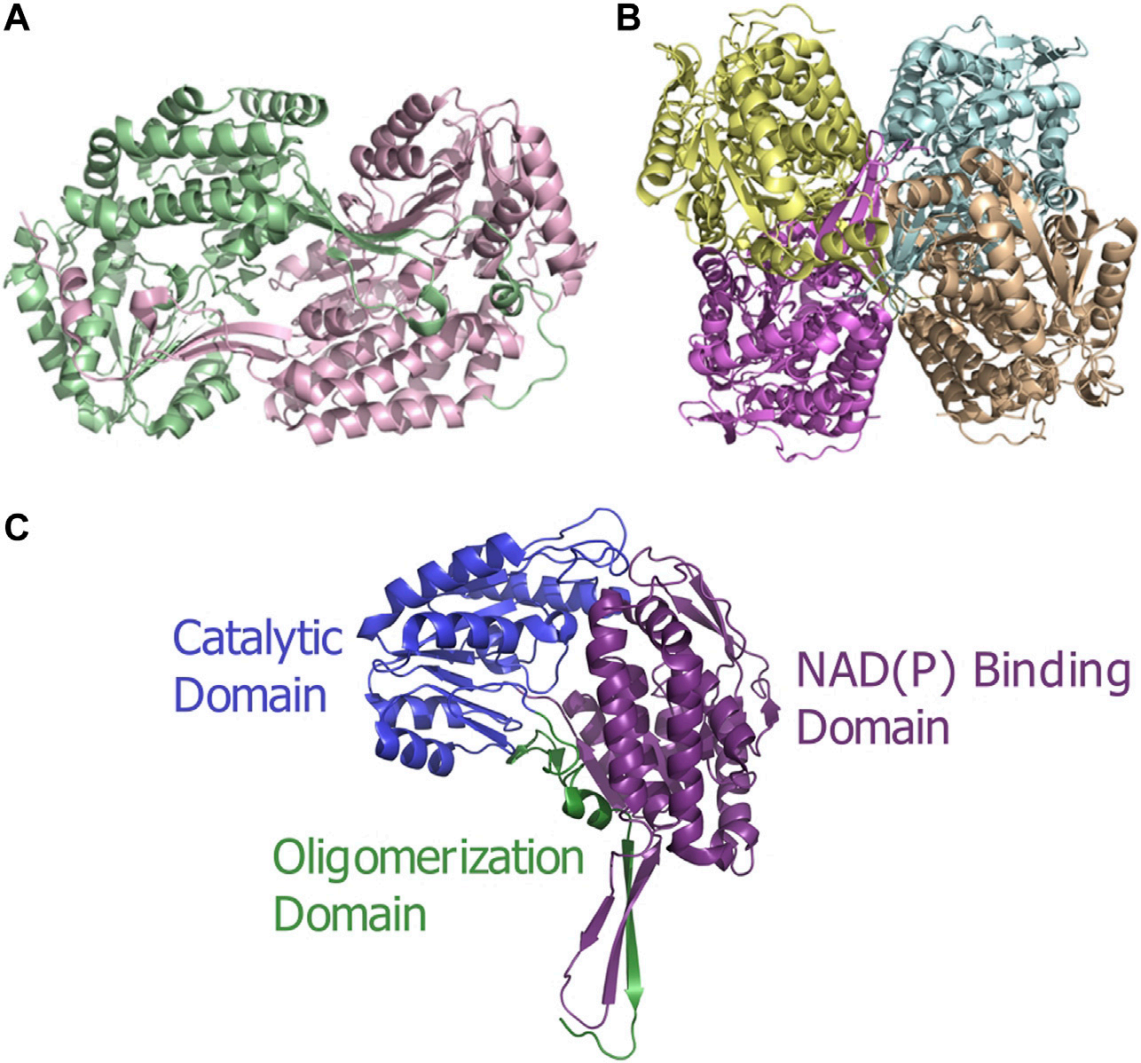
ALDH architecture **(A)** Homodimeric structure of the human ALDH3A1 (PDB: 3SZA). **(B)** Homotetrameric structure of the human mitochondrial ALDH (PDB: 1NZX). **(C)** Three conserved domains illustrated on an ALDH monomer. The functional domains are highlighted: catalytic domain (blue), NAD(P) binding domain (purple), and oligomerization domain (green).

ALDH enzymes typically exist and function as homotetramers or homodimers (Figures 1A,B) formed by subunits of approximately 450–500 amino acids to construct a 50–60 kDa protomer. The overall structure consists of three distinct, conserved domains, the NAD(P) binding domain, the catalytic domain and the “arm-like” oligomerization domain Liu et al. (1997) establishing a domain fingerprint for this superfamily (Figure 1C). At the interface of these domains, buried within the enzyme, is a funnel-shaped cavity with an opening leading to the catalytic pocket. Which within harbors the important catalytic thiol, Cys residue.

### The Active Site

The active site of the ALDH is located at the base of a hydrophobic, funnel shaped tunnel, close to the subunit’s interface and opposite the cofactor binding site. A highly reactive active-site cysteine residue (Cys302 in ALDH1a1 and ALDH2, Cys243 in ALDH3a1, human numbering), which is highly conserved throughout the ALDH family, has been accepted as the catalytic thiol (Farres et al., 1995). The side chain of the Cys residue protrudes into the catalytic tunnel that extends through each subunit. The upper portion of the funnel between the catalytic Cys and the entrance, is made up of residues from all three domains. Furthermore, this passage is identified as a catalytic pocket, relating to where the aldehyde first forms a tetrahedral intermediate with a Cys residue to initiate catalysis. In addition to aldehyde oxidation, some ALDHs possess NAD esterase activity (Feldman and Weiner, 1972; Sládek, 2003; Vasiliou and Nebert, 2005). Interestingly these enzymes utilise the same active site residues to carry out this function as dehydrogenase catalysis, however esterase activity does not require the addition of the NAD(P) cofactor (Koppaka et al., 2012). The so-called “pseudoenzyme” human ALDH16 lacks this catalytic Cys residue, resulting in absence of catalytic activity, proposed to function as a binding protein (Liu and Tanner, 2019).

Conserved residues are present across the sequences of the ALDH members, highlighting vital, positional requirements for systematic catalysis by the enzyme. Sequence alignment of 145 known ALDHs demonstrated 4 invariant residues and 12 highly conserved residues (present in 95% of sequences analyzed). Of these, glycines and prolines were abundant (7 and 2, respectively), likely highlighting critical chain-bending points due to the anti-helical and beta-sheet potentials of these residues (Perozich et al., 1999b). The catalytic Cys is conserved in all structures which harbor catalytic activity, the above mentioned ALDH16 being a prime example of a non-catalytic member (Liu and Tanner, 2019). In addition, two other important conserved residues associated with catalysis, lysine (Lys192) and glutamic acid (Glu268) (human numbering), are evident across the ALDHs. Glu268 is directly involved in catalysis providing a water molecule required for deprotonation of the catalytic thiol and subsequent hydrolysis of the thioester intermediate (see details at the end of the section). Glu399 is also highly conserved and is involved in NAD binding along with Lys192, however, Glu399 is not as critical as Lys192, because during catalysis it is bound to the nicotinamide ring which appears to have to move during the catalytic process (discussed further below). An amino acid exchange of Lys192 causes an alteration in NAD binding and the rate-limiting step but substitution of Glu399 only alters the latter (Sheikh et al., 1997).

**FIGURE 2:**
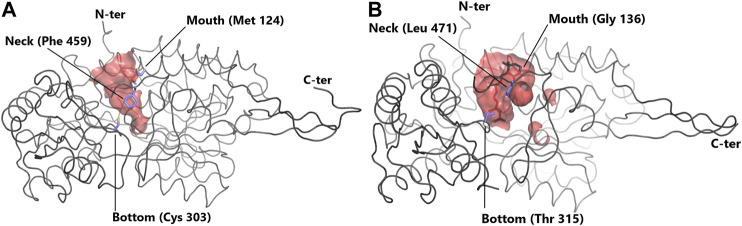
Substrate entry channel (SEC) of ALDH. Surface representation of the SEC of **(A)** ALDH2 (PDB: 3N80) showing a narrow channel suitable for small aldehydes and **(B)** of the larger SEC of ALDH1A3 (PDB 5FHZ) with retinoic acid. SEC signature residues are shown in sticks. For clarity, residues 436–456 (ALDH2) and 437–466 (ALDH1A3) are not shown.

Contrasts in ALDH enzymes exist within the cavity used for entry of the substrate into the active site, called the substrate entry channel (SEC) (Sobreira et al., 2011). The size and shape of the SEC dictates the ability of the ALDH to process small or large aldehydes (Figure 2). Typically, ALDH1 has a larger SEC volume than ALDH2 (589 ± 59 and 403 ± 53 Å^3^, respectively), consistent with their favored substrate. Human ALDH2 displays a narrow channel with a constricted entrance Steinmetz et al. (1997), one of its main substrates being acetaldehyde. In contrast sheep ALDH1A1 exhibits a large SEC equipped with a broad opening enabling access for larger aldehydes (Moore et al., 1998). This highlights that SEC topology influences substrate preferences within the ALDH family. ALDH2, with acetaldehyde as its natural substrate, displays no activity for retinaldehyde, while ALDH1 can oxidise retinaldehyde but can only inefficiently process acetaldehyde (Klyosov, 1996; Moore et al., 1998). Three key signature residues present in the SEC are responsible for geometry/function and thus substrate specificity. These residues are designated the “mouth” (124), “neck” (459) and “bottom” (303) (human ALDH2 numbering) (Figure 2) (Moore et al., 1998; Sobreira et al., 2011). The “mouth” residue performs a size selection function, bovine ALDH2 possesses a bulky Met and sheep ALDH1A1 and human ALDH1A3 a Gly (Moore et al., 1998)(Steinmetz et al., 1997; Moretti et al., 2016), resulting in either an open or narrow entrance to the SEC. The “neck” residue is present at the proximal third of the channel. In vertebrate ALDH2 this residue is a large Phe in contrast to ALDH1 where it is typically a smaller Val or Leu (Sobreira et al., 2011)(Moretti et al., 2016). The third signature, the “bottom”, is located directly beside the catalytic Cys at the end of the channel. In vertebrate ALDH2s this residue is usually a Cys, whereas in ALDH1 it can be Thr, Ile or Val (Sobreira et al., 2011; Moretti et al., 2016). Within ALDH2 the “neck” and “bottom” residues (Phe459 and Cys303) assist in holding the smaller aldehyde substrate close to the catalytic Cys–a mechanism not required for the larger substrates in ALDH1. Favourable surface interactions between Cys303 (“bottom”) and Phe459 (“neck”), fixates the position of the “neck” residue and in turn the position of Phe465 which is responsible for holding the substrate close to the catalytic thiol. This mechanism is not present in ALDH1 due to the residues substitutions as highlighted above, further emphasising the specialisation of the SEC for preferred substrates (Sobreira et al., 2011). This highlights that the size of the key amino acid signatures dictate substrate specificity while also playing a key role in surface interactions for further SEC specialization. Interestingly it was demonstrated that metazoan class 1 and class 2 ALDHs are members of a single clade, with ALDH2 forming a subgroup nested within this ALDH1/2 clade. Throughout evolution, class 1 and 2 often switched between small and large SECs following gene duplication, transforming restricted small channels into wider ones and vice versa. Expansion of the channel occurred due to substitution of a bulky methionine residue with a small alanine or glycine, reducing steric hindrance effects (Sobreira et al., 2011).

The electrostatic potential of the SEC plays a significant role in dictating the range of substrates which can be utilised for oxidation. The binding site and channel are tailored, optimising the processing of certain aldehyde substrates. ALDHs that bind positive substrates, commonly display a negative electrostatic potential in the SEC and vice versa. In contrast, ALDHs that have non-polar substrates contain neutral electrostatic potential surfaces for easy, efficient conversion (Riveros-Rosas et al., 2013). For example, human ALDH1, with a physiological substrate, retinaldehyde, has a neutral SEC (Riveros-Rosas et al., 2013; Moretti et al., 2016). ALDHs who use positive betaine aldehydes as their substrates e.g., ALDH9 from *Pseudomonas eruginosa* (PDB: 2WME) and ALDH25 from *Staphylococcus aureus* (PDB: 4MPB) are equipped with negatively charged SECs (González-Segura et al., 2009; Riveros-Rosas et al., 2013; C. Chen et al., 2014). In addition, ALDH11 from *Streptococcus mutans* (PDB: 1EUH) uses glyceraldehyde-3-phosphate as its substrate and displays a positive electrostatic potential at the SEC (D'Ambrosio et al., 2006). In contrast human ALDH3 (PDB: 3SZB), demonstrating a negatively charged SEC is known to convert medium to long chain aliphatic and aromatic aldehydes raising the question of the possibility of an unidentified positively charged substrate for this enzyme (Muzio et al., 2012; Riveros-Rosas et al., 2013).

**FIGURE 3:**
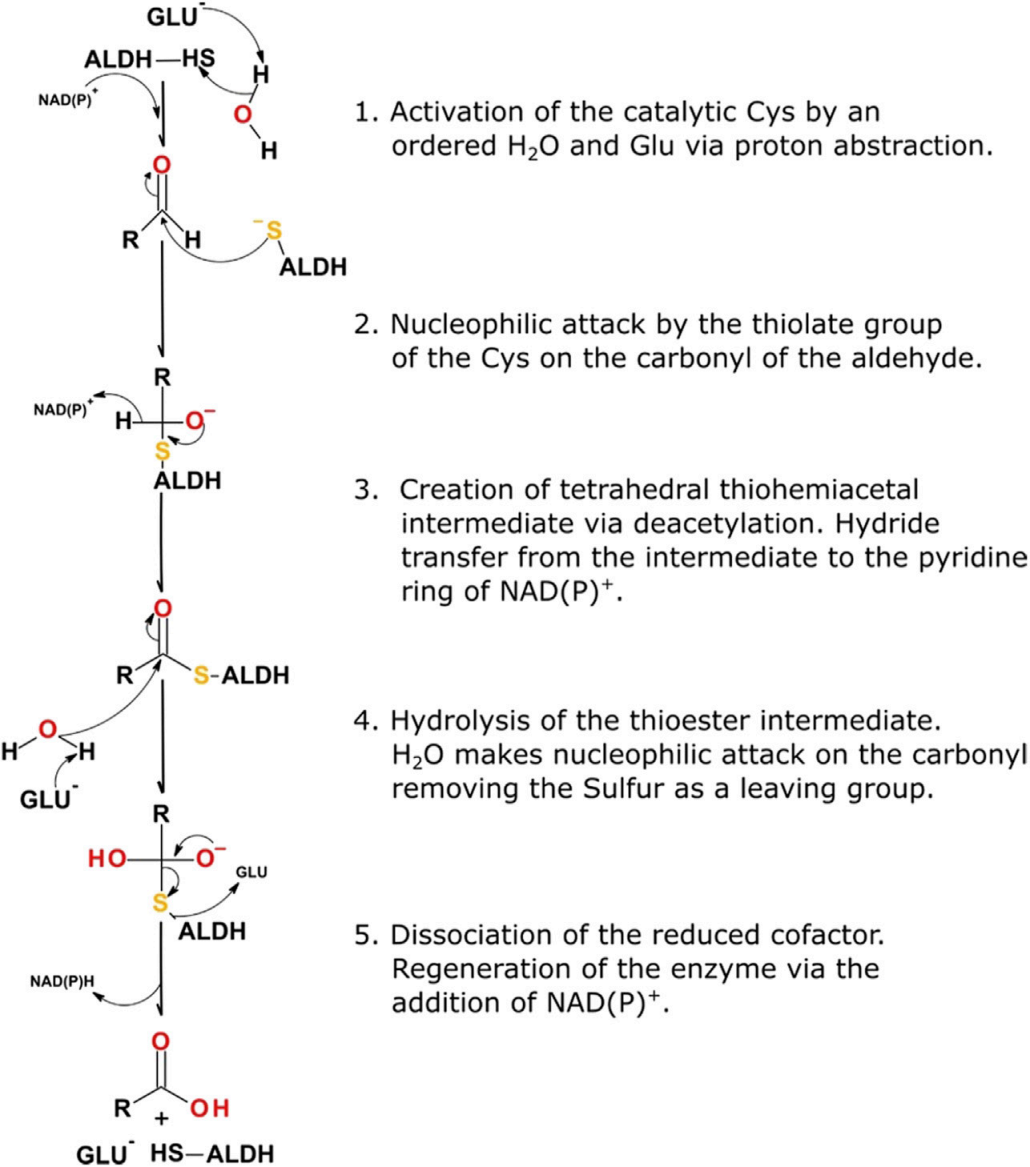
ALDH reaction mechanism highlighting the five essential steps in the catalytic scheme.

Systematic catalysis performed by an ALDH occurs in five distinct steps within the active site, 1) activation of the catalytic thiol, Cys302 or equivalent, using a water molecule for water-mediated deprotonation by Glu268, 2) consequential nucleophilic attack on the electrophilic aldehyde by the thiol group of the catalytic cysteine, 3) formation of a tetrahedral thiohemiacetal intermediate, via deacylation, coupled with concomitant hydride transfer to the pyridine ring of NAD(P), 4) hydrolysis of the resulting thioester, 5) dissociation of the reduced cofactor producing NAD(P)H and regeneration of the enzyme by NAD(P) binding (Figure 3). It is proposed that an ordered water molecule plays an essential role in facilitating catalysis. This water molecule is required to be bound to the side chain of Glu268 to allow for both the deprotonation of the catalytic thiol and subsequent hydrolysis of the thioester intermediate (steps one and four highlighted above) (Koppaka et al., 2012).

### NAD(P) Cofactor Choice and Utilisation

**FIGURE 4:**
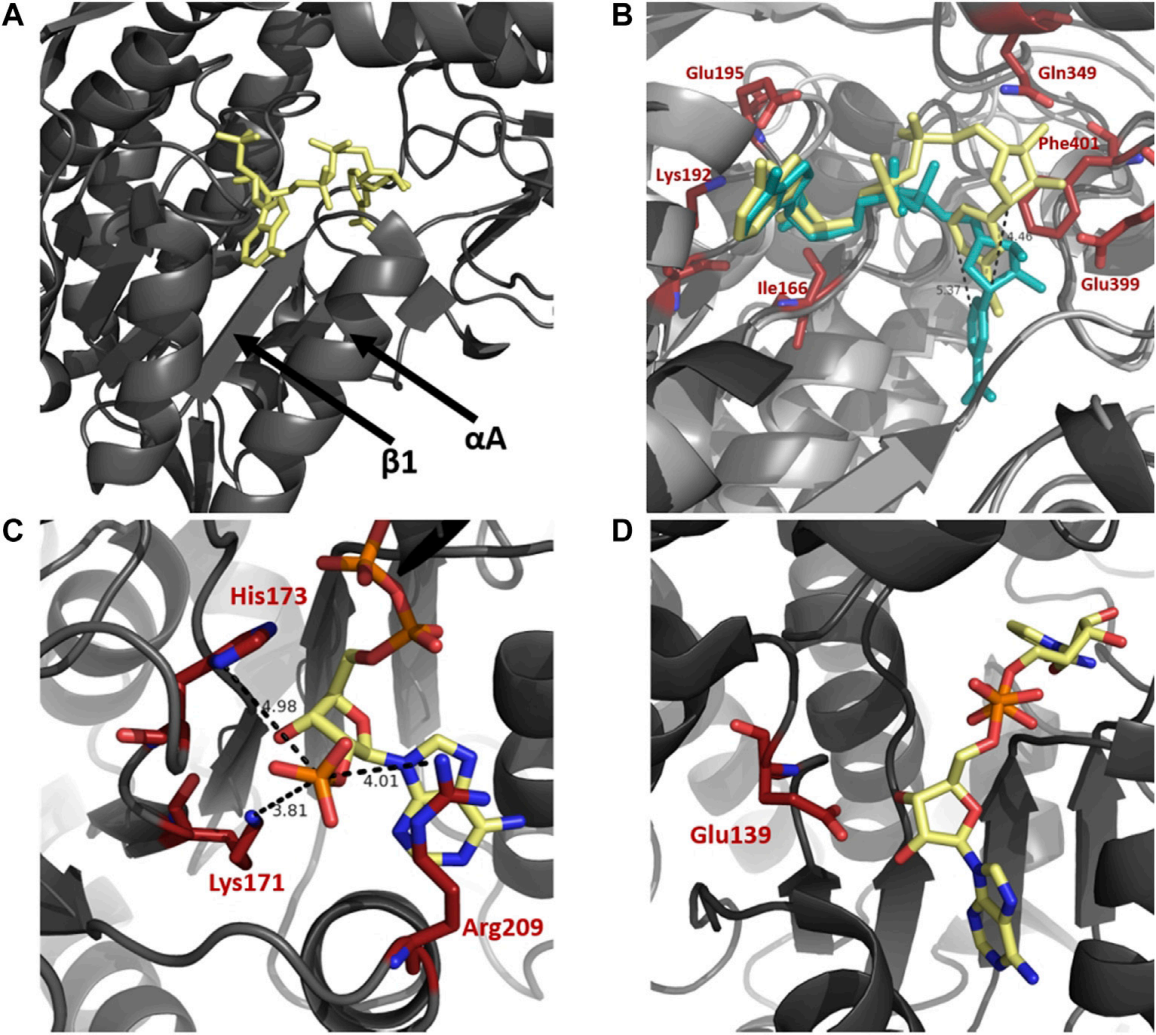
Cofactor binding mechanism of ALDH. **(A)** Depiction of αA and β1 of the Rossmann fold in the cofactor binding domain. **(B)** Varying conformations of the nicotinamide portion and constant orientation of adenine ring of NAD demonstrated on sheep ALDH1 and bovine ALDH2 (PDB: 1BXS and 1A4Z). ALDH1 (dark gray) and ALDH2 (light gray) are modeled as an overlay with NAD in yellow and blue, respectively. Measurements demonstrate an approximate 5 Å shift of the nicotinamide portion. Key cofactor binding residues are highlighted in red. Residues 241–253 have been omitted for visualisation purposes. **(C)** NADP 2′phophate shown in close proximity to Lys171, His173, and Arg209, no acidic residue is present in the NADP dependent ALDH from *V. Harvei* (PDB: 1EYY). **(D)** Human ALDH3A1 (PDB: 4L2O), a non-obligatory NADP ALDH, shows a NAD cofactor in close proximity to Glu139 even though this ALDH can utilise NADP.

A selection of enzymes use dinucleotide cofactors, such as NAD or flavin adenine dinucleotide (FAD), and even though their overall enzyme structures may differ they are normally equipped with dinucleotide binding domains consisting of either a Rossmann fold (Rossmann et al., 1974; Wierenga et al., 1986) or a (α/β)_8_ barrel structure (Wilson et al., 1992; Hoog et al., 1994). The cofactor-binding domain in ALDH is composed of a Rossmann fold. The pyrophosphate moiety of the cofactor makes close contact with the first β-α-β-α-β of the Rossmann fold, specifically the loop between β1 and αA (Figure 4A). The helix αA has been termed the “dinucleotide binding helix” (Hol et al., 1978) due to the helix dipole providing a favourable interaction with the negatively charged pyrophosphate moiety of the dinucleotide molecule (Liu et al., 1997).

In NAD binding, binding of the adenine part of the cofactor is conserved across ALDHs in a fixed conformation, with no movement necessary during catalysis. In contrast, the nicotinamide portion appears to be flexible, contributing to variable conformations throughout superfamily members (Figure 4B). Movement of the nicotinamide in and out of the active site during catalysis is a conserved and important mechanism of functional ALDHs. The adenine ring of the NAD(P) molecule during binding resides in a hydrophobic pocket between the helices, αF and αG, as observed in structures of sheep ALDH1, bovine ALDH2 and rat ALDH3 (Liu et al., 1997; Steinmetz et al., 1997; Moore et al., 1998). Stabilisation of the adenine ring within the enzyme is conferred by cradling of the ring using residue sidechains and hydrogen bonds. In both ALDH1 and ALDH2 structures the adenine ribose forms hydrogen bonds with Lys192, Glu195 and Ile166. The specific amino acid sequence, G_1_XG_2_XXG_3_, which reflects the turn at the end of the first β-strand, interacts with the adenine ribose of the NAD molecule (Wierenga and Hol, 1983; Hempel et al., 1993). The nicotinamide portion of the cofactor seemingly demonstrates discrete disorder during binding leading to a number of cofactor conformations. In contrast to bovine ALDH2 and rat ALDH3 the nicotinamide ring binding in sheep ALDH1 was different (Moore et al., 1998). Two major conformations of the nicotinamide half were observed however with the less occupied conformation mimicking bovine ALDH2 structure. The specific differences between the ALDH1 and ALDH2 are realised, the nicotinamide ring of ALDH1 occupies nearly the same position as the nicotinamide ribose in ALDH2, a shift of over 5 Å (Figure 4B). In addition, the same amino acids, Gln349, Glu399 and Phe401, confer the nicotinamide portion stabilisation in both ALDH1 and ALDH2 however they do so in a very different manner. Flexibility of the nicotinamide is also assisted by the intentionally weak binding of the pyrophosphate moiety in ALDHs. In contrast to other NAD-dependent dehydrogenases there are few interactions between the negative phosphates and the protein residues, especially due to lacking lysines or arginines in the pocket. Instead, the interactions occur with a patch of strong negative electrostatic potential near the phosphate binding pocket conserved in ALDH1 and ALDH2. These interactions act like a ball and socket joint conferring flexibility within the molecule. The rational for nicotinamide flexibility requirements relate to the water mediated deprotonation using Glu268 during catalysis, with the Glu268 also exhibiting disorder. Specifically, during the hydride transfer step the side chain of Glu268 must be tucked away from the nicotinamide ring of the cofactor. Before deacylation of the thioester can occur, the nicotinamide portion must at least half exit the active site to allow for room for the water molecule to position itself near the thioester carbon of the acyl intermediate. Flexibility and variable conformations of both Glu268 and the nicotinamide ring are paramount for proper dehydrogenase action by ALDH.

The specificity of the enzyme to utilise a sole cofactor is dictated by distinct features within the cofactor-binding domain. The Rossmann fold commonly contains an acidic residue located at the end of β2, which seemingly dictates cofactor preference. ALDH enzymes that favor the use of NADP may possess positively charged amino acids that stabilise the negative 2′ phosphate moiety. Absence of an acidic residue may prove essential for preference of NADP, as if present, repulsion effects would occur between the two negative charges (Perozich et al., 2000). The presence of an acidic residue in close proximity to the position of 2′ phosphate may dictate the cofactor specificity as it does not allow NADP to be stable within the active site, deeming the enzyme preferable to NAD (Sharkey et al., 2013). From structural analysis, human ALDH2 and ALDH from *Thermus thermophilus* (TtALDH_530_) both possess a glutamic acid residue in close proximity to the 2’ phosphate when using NADP, while both enzymes prefer NAD. ALDH from *Vibrio Harveyi* and *Pyrobaculum* sp*.* both lack a Glu residue, with *V. Harveyi* possessing an adjacent lysine residue, both utilise NADP preferably (Figure 4C). In contrast human ALDH3A1 and ALDH3A2 can use NADP but are not obligatory NADP enzymes. They are shown to possess the corresponding acidic, glutamate residue at the correct position despite their use of NADP as a cofactor (Sharkey et al., 2013) (Figure 4D).

### Structural Analysis of Oligomerization State: Dimer vs. Tetramer vs. Hexamer

**FIGURE 5:**
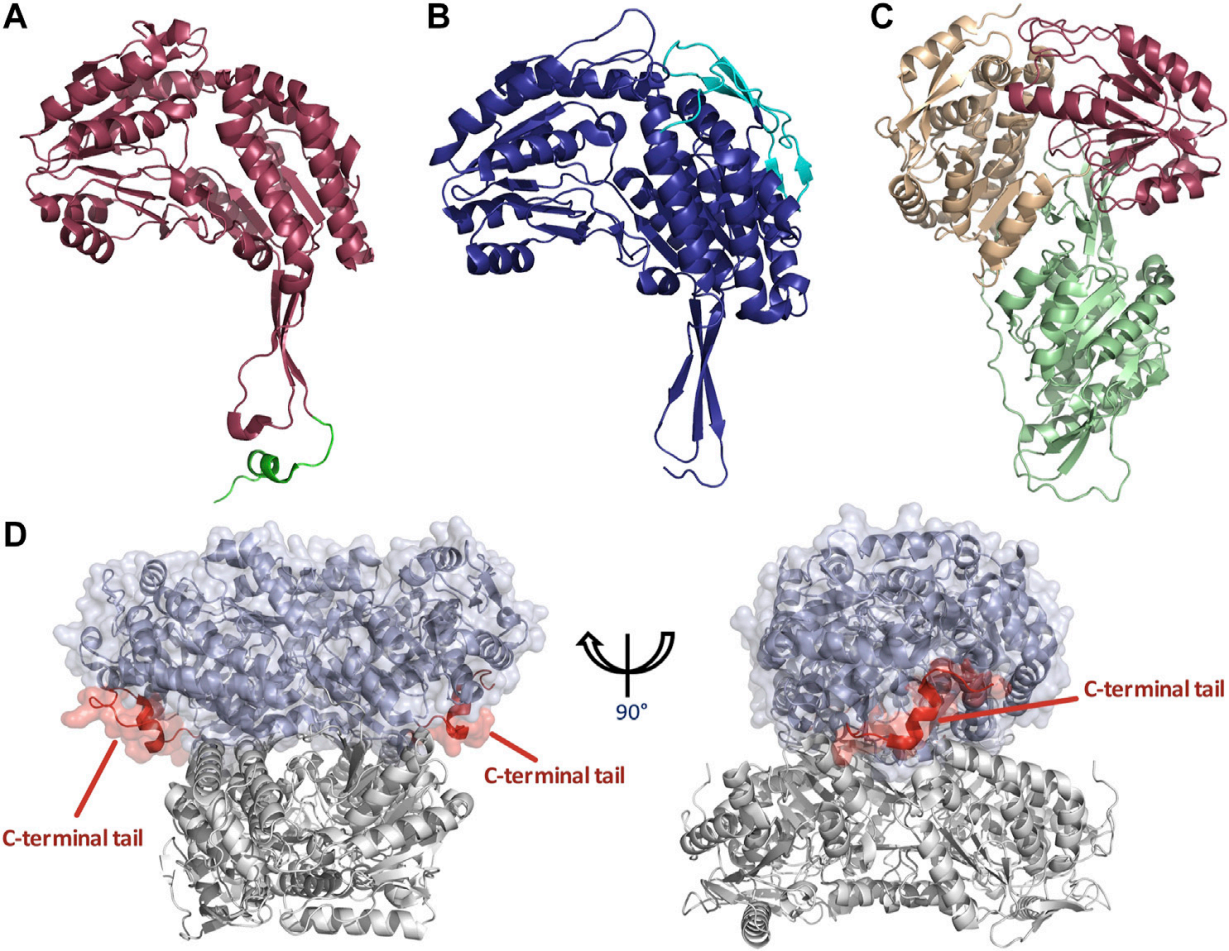
Structural features of dimeric and tetrameric ALDHs. **(A)** The C-terminal tail extension of dimeric ALDHs is represented in green against the monomer of ALDH3A1 (Red, PDB: 3SZA). Note the deletion of the first 56 amino acids in comparison to image (B) of the tetrameric ALDH2 (cyan). **(B)** The “so-called” N-terminal extension is represented in cyan against the monomer of ALDH2 (Blue, PDB: 1NZX). Note the absence of the C-terminal extended tail. **(C)** LsALDH16 monomer highlighting the NAD binding domain (orange), the catalytic domain (red) and an extra structural domain (green). **(D)** Surface representation of the dimeric ALDH3A1 (light blue, PDB: 3SZA) superimposed on ALDH2 (Gray, PDB: 1NZX). The C-terminal tail of ALDH3A1 is depicted in red. For clarity, only the opposing dimer of ALDH2 is shown.

**FIGURE 6:**
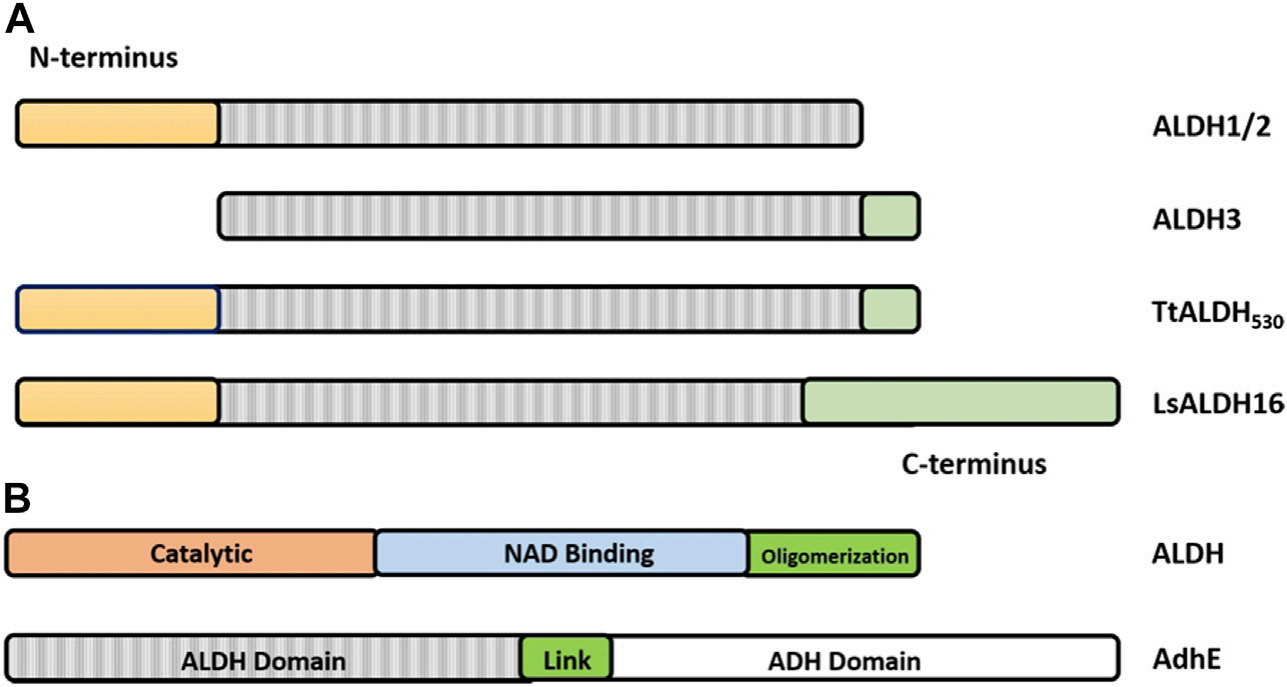
Graphical representation of the architecture of ALDHs. **(A)** ALDH class 1 and 2 being defined by a set of N-terminal residues (yellow) and the lack of the C-terminal tail (green). ALDH class 3 being defined by the absence of N-terminal residues and presence of the C-terminal extension. Note that the TtALDH_530_ contains both this N-terminal segment and the extended C-terminus with LsALDH16 containing a C-terminus constructed by a non-functional Rossmann fold domain. Modified from Hayes, et al. (Hayes et al., 2018). **(B)** Representation of ALDH functional domains on a typical ALDH and AdhE spirosome of 3 subunits. Note: size of the graphic representation does not directly relate to the size of the domains.

As previously stated ALDHs exist in dimeric or tetrameric state, but a small number of resolved structures have now demonstrated the presence of hexameric enzymes. ALDHs are constructed by domain swapped dimerization with tetramers typically being formed by a dimer-of-dimers (Figure 1). A resounding question within the literature asks, what dictates the oligomeric state which an ALDH enzyme adopts? In dimeric ALDHs, an extension of approximately 17 amino acids at the C-terminus in the form of a tail occurs. When compared to their tetrameric counterparts a 56 amino acid deletion at the N-terminus is present (Figures 5A,B, 6A). It has been tacitly assumed that the presence or absence of a C-terminal tail within the enzyme dictates the oligomeric mode, between a dimer or a tetramer. Indeed, in the dimeric structures the C-terminal tail extends into the region where a second dimer pair would assemble and, thus, disrupting tetramer formation (Hurley et al., 1999; Rodriguez-Zavala and Weiner, 2001). Figure 5D demonstrates the position of the dimeric ALDH3 C-terminal tail in comparison to a tetrameric ALDH2 organisation. Here it is demonstrated that the orientation of the tail is modeled toward a region to the outer sides of the dimer. Studies which eliminated the C-terminal tail from the dimeric structure demonstrated that a tetramer was formed at low salt conditions but reverted back to a dimer upon increase of ionic strength (Rodriguez-Zavala and Weiner, 2001). Also, the addition of 5 and 17 amino acids at the C-terminus of a tetrameric isozyme subunit was trialled, but conversion to a dimer was not achievable. When 5 residues were added, tetrameric structure was conserved but activity was reduced to 30% compared to wild type. When 17 residues were added this greatly decreased the stability of the enzyme (Rodriguez-Zavala and Weiner, 2001). This demonstrates that the C-terminal tail contributes to quaternary arrangement in dimeric structures but does not affect tetrameric ones. However, the tail is not the sole factor holding the dimer together, as upon removal and high ionic strength the dimer was conserved, suggesting other favourable interactions such as between single residues or inter-domain interactions.

**FIGURE 7:**
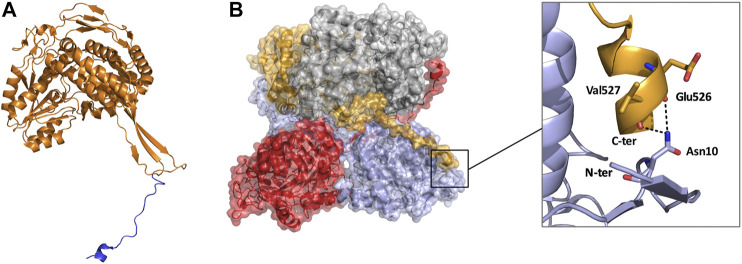
Monomeric and tetrameric structure of TtALDH_530_ (PDB: 6FJX). **(A)** Monomer highlighting the C-terminal extension in blue. **(B)** Surface representation of the tetrameric assembly, note how the C-terminal tail from one monomer wraps its diagonal monomer and interacts with its N-terminus. Monomers are represented in contrasting colors.

Until recently all tetrameric ALDHs were organized as dimer of dimers free of C-terminal extensions. However, emerging research suggests the contrary. The novel ALDH from *T. thermophilus* HB27 (TtALDH_530_) Hayes et al. (2018) showed an interesting feature with an unusual extended C-terminal tail compared to available structures (Figure 7). In contrast to other ALDHs, this extended tail contributes to the tetrameric assembly and the stability of the protein as it completely wraps the opposing monomer. This results in the formation of a network of salt bridges and hydrogen bonds with the N-terminal residues and oligomerization domain of the opposite monomer. As the tail wraps the opposing monomer, it is dragged across the opening of the substrate entry tunnel conferring possible roles in active site regulation. In addition, an ALDH from *Pseudomonas putida* (PpALDH) contains an extension in its oligomerization domain associated with “hugging” its neighboring monomer and interacting with its active site (Crabo et al., 2017). This enzyme contains an additional 6 residues in comparison to its closest related homolog, the sheep liver ALDH1, and is associated with occluding active site entrance perhaps conferring substrate specificity. This enzyme ultimately preferred smaller aldehyde substrates. Similarly, the recently resolved structure of an ALDH16 from *Loktanella* sp. (LsALDH16) demonstrated a unique oligomerization mode and potential regulation of catalysis utilising a C-terminal extension (Liu and Tanner, 2019). ALDH16 shows what has been described as transhierarchical structural similarity, where tertiary interactions within one protein mimic quarternary interactions in another. Indeed, ALDH16 features the classical NAD and catalytic domains in addition to a large non-functional cofactor binding domain in the C-terminal (Figures 5C, 6A). In this case the overall structure of LsALDH16 mimics the classical tetrameric organisation, although being formed by only two subunits.

The first description of a membrane associated ALDH, the dimeric fatty aldehyde dehydrogenase (FALDH), displayed an unrecognised C-terminal, hydrophobic “gatekeeper” helix with a function in active site restriction and substrate specificity (Keller et al., 2014). The helix induces a 93° kink toward the SEC. Adjacent is a transmembrane helix which typically anchors the FALDH to the endoplasmic reticulum or peroxisomes assisting in processing of long chain aldehydes generated in the membrane which are not accessible to cytosolic enzymes. Once the FALDH is embedded in the membrane the active site funnel and “gatekeepers” are oriented toward the membrane, with the cofactor domain toward the cytosol. “Gatekeeper” residues form a hydrophobic ring around the entrance to the SEC, possibly allowing for entry or fusion with membranes to facilitate efficient substrate binding. Removal of the gatekeeper helix resulted in reduced capacity for hexadecanal but not toward shorter aldehydes such as octanal (Keller et al., 2014). Recently a similar C-terminal “gatekeeper” was observed in the *Staphlococcus aureus* ALDH (SaALDH) but with the characteristic kink orientating the helix away from the SEC (Tao et al., 2020). Upon binding of substrate a conformational change associated with the C-helix is adopted, a change in direction of the kink by 10.6° now orients the “gatekeeper” toward the SEC. Without substrate, in SaALDH, the C-helix takes an open conformation allowing for substrates to enter, whereas with bound substrate the C-helix locks the aldehyde in place and adopts a closed conformation.

**FIGURE 8:**
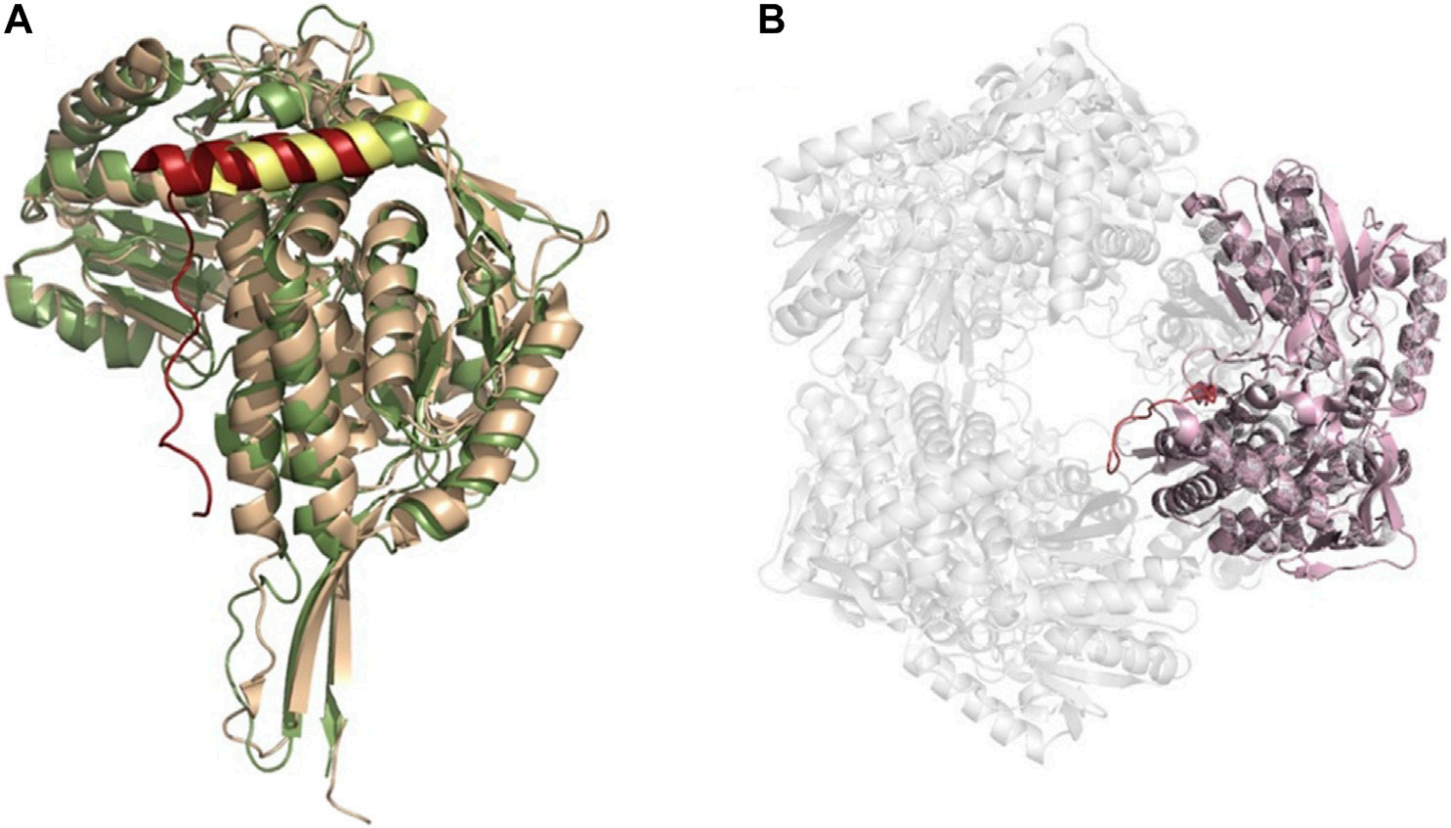
ALDH hexameric structure **(A)** Superimposition of the monomers of yeast ALDH4A1 (orange) and TtP5CDH (green) (PDB: 4OE6, 2BHQ, respectively), demonstrating the N-terminal extension equipped with an alpha helix in hexamer forming ALDHs. Extension is shown in red for TtP5CDH and yellow for yeast. **(B)** The hexameric assembly of TtP5CDH (PDB: 2BHQ) with the N-terminal loop (red) penetrating the pore formed by the hexamer.

A third oligomeric state of ALDHs exists in the form of a hexamer (Figure 8), organized as trimer of dimers. However, this is a less common state and there are only two resolved structures from *S. cerevisiae* (ScALDH4A1, 4OE6) and *T. thermophilus* (TtP5CDH, pyrroline-5-carboxylate dehydrogenase, 2BHQ), with another unavailable structure from *Deinococcus radiodurans* being reported (Inagaki et al., 2006; Luo et al., 2013; Pemberton et al., 2014). No mammalian hexameric structures have been reported to date. Work on TtP5CDH showed that the formation of the hexamer is mainly associated with the presence of a hexamerization hotspot. An arginine residue was identified in the bacterial model while a tryptophan residue assumes this role in ScALDH4A1 (Pemberton et al., 2014). Without this essential hotspot residue, hexamer formation is compromised (Luo et al., 2013). Interestingly, through our own structural analysis, a unique feature of the hexamer structure was identified. In TtP5CDH, a 33 amino acid extension of the N-terminus is evident, consisting of a loop of 15 amino acids penetrating the pore formed by the hexameric assembly and interacting with the adjacent subunit, while the remaining 18 residues are organized in an alpha helix. In comparison, the yeast ScALDH4A1 shows a similar alpha helix, however, with the first 30 amino acids not visible in the structure due to disorder (Figure 8).

### Alcohol Dehydrogenase-Aldehyde Dehydrogenase Bifunctional Spirosomes

**FIGURE 9:**
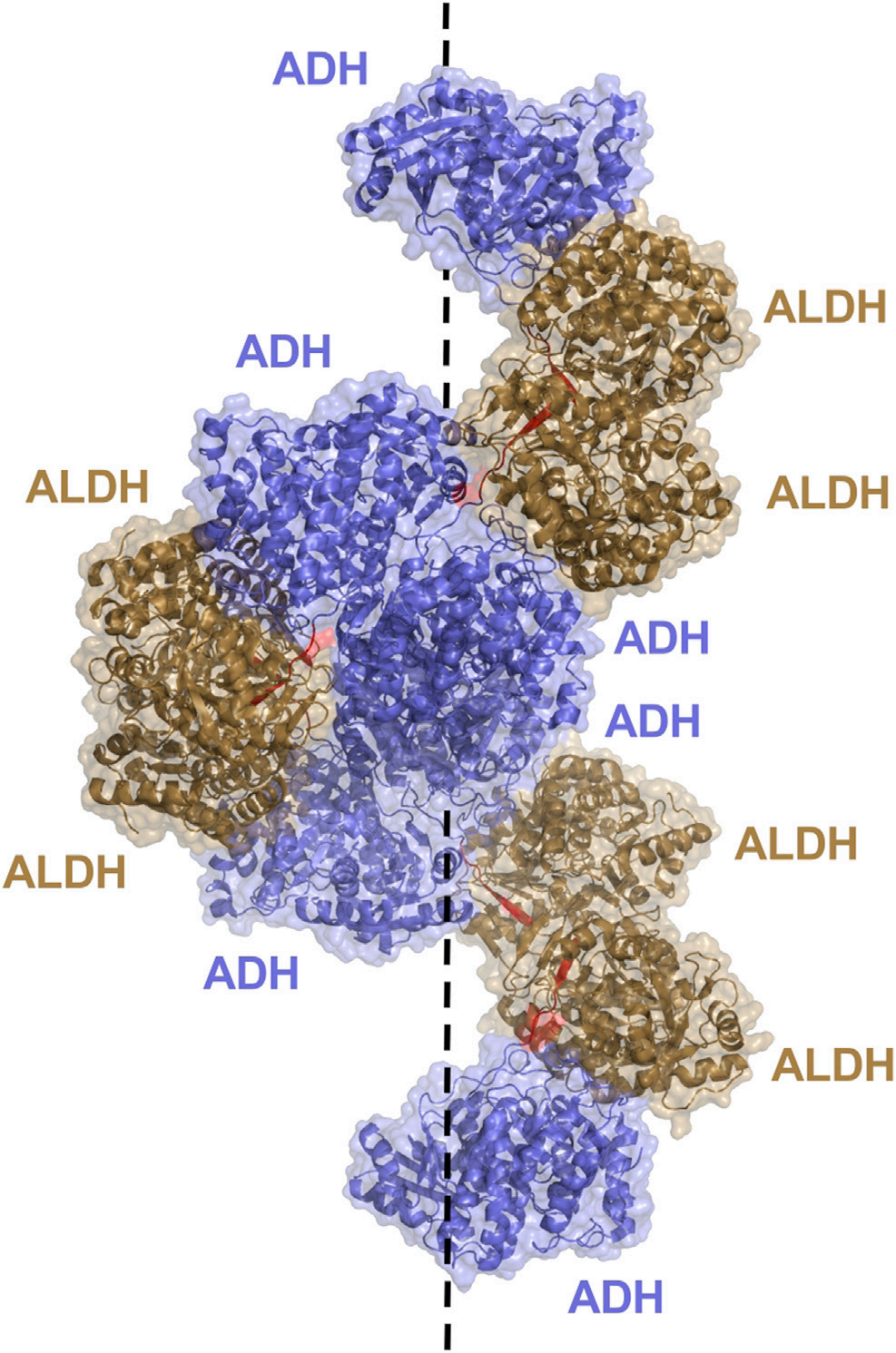
AdhE extended spirosome structure shown as a cartoon with transparent surface representation (PBD: 7BVP). ALDH domains located on the outer surface are shown in brown, ADH domains in blue on the inside of the spirosome and the 7 amino acid linker between domains in red. The dotted line demonstrates the helical axis of the spirosome.

Aldehyde-alcohol dehydrogenase (AdhE) is a bifunctional enzyme, key to bacterial metabolic processes in physiology and pathology, which contains two enzyme domains, ALDH and alcohol dehydrogenase (ADH). While monofunctional ALDH and ADH are found in all kingdoms of life, AdhE is mostly found in bacteria and some unicellular eukaryotes such as microalgae. These helical macromolecule assemblies, called spirosomes, were first discovered in 1975 Kawata et al. (1975) and were more recently designated as filaments (Pony et al., 2020). A recent, in depth study of the *E. coli* AdhE was carried out by Kim et al. highlighting their critical structure-function relationship (Kim et al., 2019). The AdhE monomer (96.1 kDa) is composed of an ALDH and ADH domain separated by a linker of 7 amino acid residues (Figure 6B). Together with the protruding β-turn from the ALDH domain the linker forms a 3 stranded β-sheet for connection of the two catalytic domains. The structures of the ALDH and ADH domains are similar to other known structures, both contain two lobes, the ALDH with a Rossmann fold and NADH binding cleft, while the ADH contains an Fe^2+^ and NADH binding pocket. Two AdhE monomers form a dimer in a head-to-head arm crossing fashion. To then form the spirosome structure, dimers are almost stacked one on top of each other, through use of hydrophobic interactions mediated by the ADH domains. For example, six AdhE molecules with two ADH domains at either end will consist of approximately 1.5 helical turns. Repetition of the helical unit leads to spirosome formation (Figure 9).

It is determined that the spirosome structure is crucial to maintain catalytic activity of the AdhE, with the ALDH and ADH activity topologically separated due to the helical filamentous structure (Kim et al., 2019). Within the structure, the ALDH and ADH domains from different subunits are clustered together rather than intertwined with mismatching domains. This allows for dividing of activity and the ability for the ALDH and ADH to work solely and more efficiently. The ALDH active site is located toward the outer surface of the helical structure, while the ADH active site resides toward the inner surface. Effective elimination of cytotoxic acetaldehyde is imagined. The acetaldehyde produced by ALDH will not enter the cell, it will be passed to the ADH within the spirosome structure for timely conversion to ethanol. Additionally, maintenance of the spirosome structure proved critical in AdhE catalytic activity. Disruption of spirosome formation and generation of free dimers through mutation led to a dramatic decrease in AdhE activity. It is notable that spirosome formation proved critical for maintenance of the forward ALDH reaction, conversion of Acetyl Co A to acetaldehyde, but no other reactions, suggesting that the spirosome might play a role in regulating the direction of AdhE activity.

The outer residing ALDH of the AdhE is exposed to solvent, whereas the ADH on the inside of the compact spirosome is not readily accessible. AdhE spirosomes undergo a structural transition in the presence of cofactor to assist with catalysis (Kim et al., 2020). A compact form is changed into an elongated, extended spirosome upon addition of cofactor (Kessler et al., 1991; Kim et al., 2019). Further suggestions lead to the cofactor binding in the ADH domain being the most critical to initiate the change. This transition may be involved in regulation of AdhE activity. Substantial structural changes upon binding of the cofactor leads to a widely opened spirosome with an accessible ADH catalytic pocket. Moreover, the transition creates a substrate channel between the ALDH and ADH active sites, from two different subunits, which is accessible to solvent and thus substrates and products (Kim et al., 2020). In this conformation the otherwise restricted ADH active site is accessible to solvent from both outside and inside, with the inter-domain channel likely playing a role for transition of intermediate products, assisting with the bifunctional cascade reaction of the spirosome.

## Structural Perspective of Aldehyde Dehydrogenase Related Diseases

ALDH enzymes are diverse in their function across all kingdoms of life, contributing to detoxification Esterbauer et al. (1991), Kelson et al. (1997), Choudhary et al. (2005), Ho and Weiner (2005), Rodríguez-Zavala et al. (2006), Marchitti et al. (2007), Shin et al. (2009), Jackson et al. (2011), Ouyang et al. (2020), biosynthesis Yoshida et al. (1992), Mellema et al. (2002), Kim et al. (2015), Pequerul et al. (2020), and non-enzymatic functions such as anti-oxidant Estey et al. (2007), Lassen et al. (2008), Marchitti et al. (2011), Singh et al. (2013), Voulgaridou et al. (2020), structural Piatigorsky (1998), Voulgaridou et al. (2017) and regulatory mechanisms Moreb (2008), Vassalli (2019), Voulgaridou et al. (2020) (Table 1). Proper function of these enzymes is essential for maintenance of cell function and survival, with prominent ALDH-linked diseases arising from mutation, catalytic knockout and structural disruption. Knowledge of the molecular and structural basis for diseases is critical for understanding pathology and ultimately for therapeutic design, typically via comparison of wild type and mutants. However, with regards to ALDH disease in literature, focus is mainly upon phenotype and not enzyme structure. A detailed breakdown of human ALDH related disease is shown in Table 2 (Chao et al., 1994; Dupé et al., 2003; Deak et al., 2005; Hui et al., 2007; Jo et al., 2007; Lassen et al., 2007; Husemoen et al., 2008; Marchitti et al., 2008; Pavan et al., 2009; Guo et al., 2010; Palo et al., 2010; Palmfeldt et al., 2011; Sulem et al., 2011; Sass et al., 2012; Vasiliou et al., 2013). Herein, we summarise and discuss previously studied ALDH diseases in context of mutations and their effect on ALDH structure and function. Disease causing mutations present in the ALDH superfamily can be arranged into three groups, these will be designated for the purpose of this review as 1) those that affect NAD binding, 2) those that affect the substrate binding site and 3) those that disrupt quarternary structure formation.

**TABLE 1:** ALDH function, categorically highlighting examples of contribution to specific mechanisms.

Biological Function		**Examples**
Catalytic	Detoxification	Removal of aldehydes, e.g., acetaldehyde, fatty aldehydes
		Fucose and phenylalanine metabolism in *E. coli*
	Biosynthesis	Retinoic acid synthesis in humans
		γ-aminobutyric acid synthesis in humans
		Osmoprotection by synthesis of betaine in prokaryotes
Non-Catalytic	Anti-oxidant	Indirect NAD(P)H generation
		UV absorption in the eye
	Structural	Corneal and lens crystallins
	Regulatory	Biomarkers and regulators of stem cells
		γ-aminobutyric acid synthesis pathway regulation
		Regulation of osmotic pressure
		Regulation of genes by retinoic acid
	Binding	Binding of endobiotics and xenobiotics
		Androgen, thyroid hormone and cholesterol binding

**TABLE 2:** Human diseases linked to ALDH genes.

**Gene**	Disease
*Aldh1a1*	Cataract formation; Parkinson’s disease
*Aldh1a2*	Disrupted embryonic development; spina bifida; rare cases of congenital heart disease
*Aldh1a3*	Disrupted embryonic development
*Aldh1b1*	Hypertension; ethanol sensitivity; ethylmalonic encephalopathy; bipolar disorder
*Aldh2*	Alcohol intolerance; heart attack; hypertension; cancers; liver cirrhosis; Parkinson’s and late onset Alzheimer’s disease
*Aldh3a1*	Cataract formation
*Aldh3a2*	Sjogren-Larsson syndrome
*Aldh4a1*	Type II hyperprolinemia
*Aldh5a1*	γ-hydroxybutyric aciduria
*Aldh6a1*	Psychomotor delay; methylmalonic aciduria
*Aldh7a1*	Pyridoxine-dependent epilepsy; osteoporosis
*Aldh16a1*	Gout
*Aldh18a1*	Hyperammonemia

**ALDH2.** The mitochondrial ALDH2, plays a significant role in ALDH-linked pathology and may be the most extensively discussed ALDH within literature in terms of disease. Specifically, the single nucleotide polymorphism E487K Farrés et al. (1994), Larson et al. (2007) residing in the oligmerization domain, resulting in the ALDH2*2 variant, is related to complications in cardiovascular disease Chen et al. (2019), cancer Seitz and Stickel (2010), alcohol intolerance Chang et al. (2017) and late onset Alzheimer’s disease (Kamino et al., 2000). ALDH2*2 can be designated as group 1 and 3 in relation to structure-function disruption. When just one protomer of the tetrameric assembly contains ALDH2*2, NAD binding is altered and catalytic activity is lost, resulting in accumulation of cytotoxic acetaldehyde (Larson et al., 2005). This mutation is responsible for a 200-fold increase in K_M_ for NAD and a 10-fold reduction in kcat, ultimately severely reducing catalytic rate *in-vivo*. Physiological levels of NAD cannot reach the demand of the ALDH2*2. Within ALDH2, the adenosine portion of NAD is nestled within the cleft present between αF and αG of the Rossmann fold. The αG helix is situated at the dimer interface and interacts with its same helix from the opposing subunit. Glu487 forms hydrogen bonds with Arg264 and Arg475 located on the C-terminus of α-G and within a loop on the opposing subunit respectively. With the E487K substitution, stabilisation of αG and the loop containing Arg475 is lost, leading to dimer destabilisation, NAD binding site disorder and some active site residue repositioning. Specifically, residues Glu399 and Glu268 essential in coenzyme positioning and catalysis respectively. A clinically relevant, specific activator of ALDH2*2 named Alda-1, was found to enhance enzyme activity *in-vitro* and *in-vivo*. Importantly Alda-1 restores activity lost by the E487K mutation, through binding at the entrance of the catalytic tunnel, dramatically decreasing K_M_ for cofactor and increasing V_max_ (Chen et al., 2008; Perez-Miller et al., 2010). E487K substitution in ALDH2 causes a loss of electron density at helix αG, and active site loop containing Arg475. As revealed by the crystal structure of Alda-1 in complex with ALDH2*2, binding of Alda-1 restores the αG structure and the loop even though Alda-1 has no direct contact with these residues (Perez-Miller et al., 2010). Alda-1 is therefore an agonist and simultaneously functions as a chemical chaperone, exerting allosteric effect to restore the structural defect of a catalytically impaired enzyme (C.-H. Chen et al., 2014).

**ALDH7A1.** ALDH7A1 is responsible for lysine catabolism and its improper function is related to a seizure causing disorder named pyridoxine-related epilepsy (PDE), linked with approx. 60 missense mutations Stenson et al. (2008); van Karnebeek et al. (2016), Coughlin et al. (2018) which are highly considered within the literature. Interestingly, the classification of the mutations led to different symptom severity and treatment options in patients. Group 1 allowed for complete seizure control and normal developmental outcome, group 2 complete seizure control but developmental delay and group 3 showed persistent seizures with developmental delay. This suggests that multimer disruption is most detrimental in terms of ALDH7A1 dysfunction. Many of the associated mutations are surface accessible suggesting a role in retention of tetrameric assembly (Scharer et al., 2010). A study investigating 6 mutations (A129P, G137V, G138V, A149E, G255D, G236E) on ALDH7A1 present at the tetrameric interface and remote from the active Cys (18–28 Å) abolished enzyme activity indirectly and disrupted tetramer formation (Korasick et al., 2017). The most common missense mutation in ALDH7A1 has been reported as E427G Coughlin et al. (2018), occurring in 30% of PDE patients Mills et al. (2006), Plecko et al. (2007), Mills et al. (2010), a mutational hotspot for disease. A study investigating structural changes of ALDH7A1, exhibiting mutations E427G, E427Q and E427D, demonstrated a catalytic defect and a non-natural conformation of the NAD cofactor and as a result no catalytic activity (Laciak et al., 2020). The NAD adopts a flexible conformation and lacks a defined pose for E427G and E427Q variants whereas an inactive pose is demonstrated for E427D, compromising catalysis due to lack of stabilisation and increased distance of the cofactor from active site Cys. In addition, this study again demonstrates the criticality of oligomerization for correct function with all three mutant variants compromised in tetramer assembly.

**FALDH/ALDH3A2.** ALDH3A2 is an endoplasmic reticulum bound FALDH responsible for the conversion of fatty aldehydes to fatty acids. Sjogren Larsson syndrome is a genetic disorder characterised by scaling skin, speech abnormalities, intellectual disability and spasticity caused by an autosomal recessive mutation in ALDH3A2, resulting in accumulation of aldehydes (Cho et al., 2018). Catalytic site mutations are mostly found in exon 4 with most exons harboring protein misfolding mutations. Interestingly the most frequent mutations, totalling 16, occur in exon 9, relating to coding for the C-terminal “gatekeeper” helix. As previously mentioned this “gatekeeper” assists in selection and easy processing of medium to long fatty aldehydes. Realised pathology could be associated with alterations in this helix as it may cause a shift in substrate specificity, inhibiting the removal of fatty aldehydes from the cell.

## Concluding Remarks

The ongoing ALDH structure-function investigation is important for elucidating the novel features of these enzymes as well as the underlying mechanism for the cause of many diseases. Advancement in recent years lead to the understanding of new oligomerization modes, domains, extensions and bifunctionality contributing to both structure and function. The evolutionary progress of these enzymes clearly shows their adaptation for tailoring of the enzyme structure for processing of defined substrates: spirosomes for efficient shuttling of substrates between two enzyme domains within one structure conferring reduced cytoxicity, hydrophobic helices for selection of fatty aldehydes as well as membrane anchorage and appropriate geometry of the SEC for processing of the correct sized aldehyde. Vast fundamental knowledge of the ALDH has been paramount for the understanding of pathological diseases caused by ALDH. Deep understanding of human ALDH catalytic mechanisms, cofactor binding and geometry of the active site and SEC has allowed for in some cases complete characterisation of disease models in terms of diagnosis, development, biochemistry and even ALDH structural mechanisms. As an ever growing superfamily of enzymes, new characteristic features will develop over time, particularly in the area of prokaryotic models. This will lead to shaping of an already well-defined family of enzymes, however adding insights and interpretation. While all members generally follow the common ALDH architecture, it is now the smaller, more niche aspects which help us develop the key understanding of these enzymes, ultimately to pick apart ALDH structure, function and disease models in a profound manner.

## Abbreviations

ADH, Alcohol dehydrogenase; AdhE, Alcohol-aldehyde dehydrogenase; ALDH, Aldehyde dehydrogenase; FAD, Flavin adenine dinucleotide; FALDH, Fatty aldehyde dehydrogenase; LsALDH16, ALDH16 from *Loktanella Sp.*; NAD(P), Nicotinamide adenine dinucleotide (phosphate); PDE, pyridoxine-related epilepsy; PpALDH, ALDH from *Pseudomonas putida*; SaALDH, ALDH from *Staphlococcus aureus*; ScALDH4A1, ALDH4A1 from *Saccharomyces cerevisiae*; SEC, Substrate entry channel; TtALDH_530_, ALDH530 from *Thermus thermophilus*; TtP5CDH, Pyrroline-5-carboxylate dehydrogenase from *Thermus thermophilus*
